# Exploring and Mobilizing the Gene Bank Biodiversity for Wheat Improvement

**DOI:** 10.1371/journal.pone.0132112

**Published:** 2015-07-15

**Authors:** Deepmala Sehgal, Prashant Vikram, Carolina Paola Sansaloni, Cynthia Ortiz, Carolina Saint Pierre, Thomas Payne, Marc Ellis, Ahmed Amri, César Daniel Petroli, Peter Wenzl, Sukhwinder Singh

**Affiliations:** 1 International Maize and Wheat Improvement Center (CIMMYT), Km. 45 Carretera México-Veracruz, Colonia El Batán, Texcoco, Edo. de México, CP, 56130, Mexico; 2 International Centre for Agricultural Research in the Dry Areas (ICARDA), Bashir El Kassar Street, Verdun, Beirut, 1108–2010, Lebanon; 3 DivSeek, Global Crop Diversity Trust, Platz der Vereinten Nationen, 53113, Bonn, Germany; National Institute of Plant Genome Research (NIPGR), INDIA

## Abstract

Identifying and mobilizing useful genetic variation from germplasm banks to breeding programs is an important strategy for sustaining crop genetic improvement. The molecular diversity of 1,423 spring bread wheat accessions representing major global production environments was investigated using high quality genotyping-by-sequencing (GBS) loci, and gene-based markers for various adaptive and quality traits. Mean diversity index (DI) estimates revealed synthetic hexaploids to be genetically more diverse (DI= 0.284) than elites (DI = 0.267) and landraces (DI = 0.245). GBS markers discovered thousands of new SNP variations in the landraces which were well known to be adapted to drought (1273 novel GBS SNPs) and heat (4473 novel GBS SNPs) stress environments. This may open new avenues for pre-breeding by enriching the elite germplasm with novel alleles for drought and heat tolerance. Furthermore, new allelic variation for vernalization and glutenin genes was also identified from 47 landraces originating from Iraq, Iran, India, Afghanistan, Pakistan, Uzbekistan and Turkmenistan. The information generated in the study has been utilized to select 200 diverse gene bank accessions to harness their potential in pre-breeding and for allele mining of candidate genes for drought and heat stress tolerance, thus channeling novel variation into breeding pipelines. This research is part of CIMMYT’s ongoing ‘Seeds of Discovery’ project visioning towards the development of high yielding wheat varieties that address future challenges from climate change.

## Introduction

Grain production needs to be doubled to feed an increasing world population which is estimated to reach approximately 9 billion by 2050 [1]. The existing trends in wheat yield increase are inadequate to meet this projected demand [2]. Bread wheat (*Triticum aestivum* subsp. *aestivum*) is one of the most important crops providing one-fifth of the total calories for the world’s population. Breeding gains rely on access to useful genetic variations from crops’ gene pools. Gene banks are the repositories of beneficial gene(s)/alleles from crop’s primary, secondary or tertiary gene pools which should be harnessed for present and future wheat genetic improvement programs [3]. Under-utilized but useful gene bank variation, when channeled into elite breeding materials using effective pre-breeding strategies, can provide diverse benefits including increased stress tolerance, yield potential and improving nutritional and processing quality [4].

During the Green Revolution era, global increases in wheat yield potential were achieved by deploying plant height genes (*Rht1* and *Rht2*; [5]), as well as numerous genes for disease resistances. The semi-dwarf, fertilizer responsive, lodging resistant and high yielding green revolution varieties replaced landraces and traditional varieties grown by the farmers [6]. As a consequence, the genetic diversity in most of the world’s wheat producing regions became limited. Even today this remains as one of the major challenges for wheat improvement [7, 8, 9] as modern high-yielding wheat cultivars possess genes or gene combinations pyramided by breeders using well-adapted cultivars. There is need to introgress new variations and gene combinations from landraces and wild species (via synthetics). In this direction, CIMMYT has enormously expanded the utilization of widely adapted germplasm which is genetically diverse, and over years have made elite gene pool almost as diverse as landraces [10, 11]. However, introgression of additional variation hidden in genetic resources is necessary to further improve wheat and to enable the continued development of high yielding cultivars which can cope well with a wide range of environmental fluctuations and stresses.

To achieve this objective, gene banks such as those at CIMMYT (International Maize and Wheat Improvement Center) and ICARDA (International Center for Agricultural Research in the Dry Areas), can play a significant role. A project currently being pursued at CIMMYT—Seeds of Discovery (SeeD; http://seedsofdiscovery.org) is centered towards characterizing and mobilizing under-utilized genetic variations from maize and wheat gene banks into breeding pipelines. Wheat accessions are being characterized for genetic diversity and phenotypic performance using the state-of-the-art genotyping and phenotyping technologies [12]. Genotyping-by-sequencing (GBS) is an advanced next generation sequencing approach for genotyping which provides a rapid, high-throughput, and cost-effective tool for performing genome-wide analysis of genetic diversity [13, 14, 15, 16]. Further, characterization of the wheat gene bank accessions for adaptive and quality trait genes has the potential to reveal novel alleles useful for breeding. Assessing genome-wide and gene-specific diversity will not only provide a robust estimate of the diversity but will also reveal the germplasm containing novel alleles which may be useful for wheat breeding programs. This will help in achieving the overarching goal to improve wheat for different environments, ecosystems and stress situations.

The present study was conducted to characterize different sets of gene bank accessions and identify useful variations that can be efficiently utilized in wheat breeding. Specific objectives of the present investigation were: (1) to quantify the molecular diversity of a set of 1,423 bread wheat accessions including specific sets of landraces assembled through a trait-based approach called focused identification of germplasm strategy (FIGS), synthetic hexaploids and elite germplasm (S1 Table) using the DArTseq-GBS approach; (2) to assess the gene-based diversity of the collection for important adaptive and quality traits; and (3) to identify novel alleles that can be deployed for wheat breeding.

## Results

### GBS diversity in different germplasm sets

DArT-based GBS SNPs was used to investigate four germplasm sets, namely, the FIGS Drought set (FD; drought tolerant landrace accessions identified through FIGS approach, received from ICARDA), the Australia Hot set (AH; landrace accessions identified as heat tolerant, received from Australian gene bank, Horsham, Victoria), synthetic hexaploids (SH) and elite lines (E). A total of 29 K GBS SNP markers were available for the FD, AH, SH, and E lines. After removing markers with missing data > 20%, minor allele frequency < 0.05 and unknown map positions, 11K markers were used for diversity analysis. S1 Fig shows GBS markers specific to each group and shared among the four germplasm groups.

**Fig 1:**
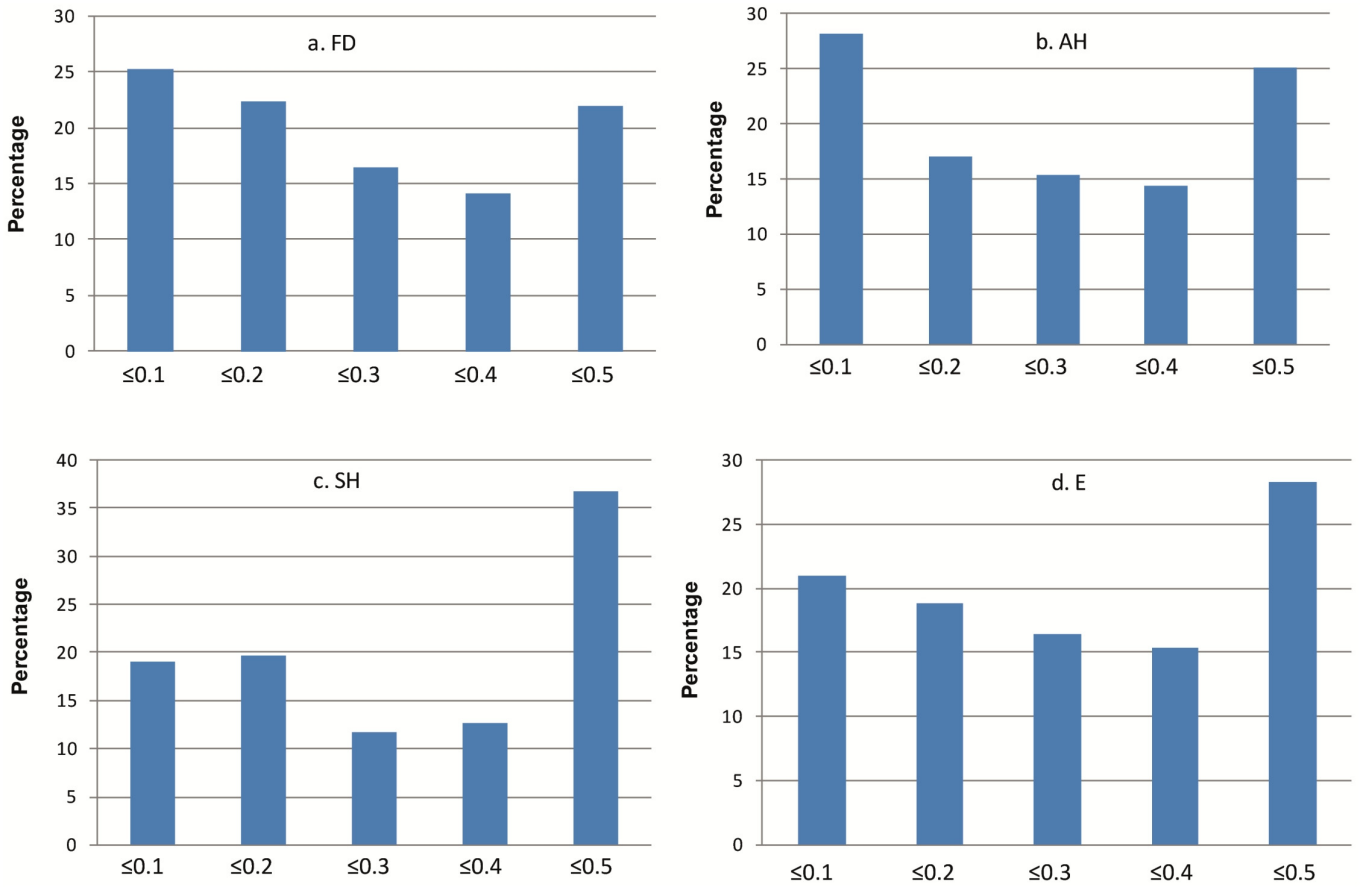
Nei’s diversity index (DI) distribution across FIGS Drought (a), Australia Hot (b), synthetic hexaploids (c) and elites (d). Each column represents percentage of markers having DI either equal to or less than the value shown on X-axis.

Nei’s diversity index (DI) was calculated for each germplasm group (Table 1). It ranged from ranged from 0.182–0.285, 0.182–0.305, 0.204–0.406 and 0.172–0.315 with mean values of 0.242, 0.248, 0.284 and 0.267 in FD, AH, SH and E, respectively. The mean within group genetic distance estimates (Table 1) in FD, AH, SH and E were 0.094, 0.105, 0.181 and 0.125, respectively. These results revealed the highest diversity in synthetic hexaploids followed by elites and landraces. To ascertain that the obtained trend is not due to sample size differences, DI was also calculated by taking an equal number of samples (211) randomly from each group, and a similar pattern of diversity was observed (S2 Table). The distribution of DIs in the germplasm sets revealed that a higher percentage of markers in both synthetic hexaploids and elite germplasm have DI between 0.4 and 0.5 as compared to landraces where maximum percentage of markers was in the group with DI ≤0.1 (Fig 1). In both landraces and elite germplasm, the D sub-genome was less diverse than the A and B sub-genomes (Figs a and b in S2 Fig), whereas in synthetic hexaploids the diversity of the D sub-genome was not only higher than its A and B sub-genomes but also the D sub-genomes of both landraces and elite lines (Table 1, Fig c in S2 Fig).

**Table 1:** Nei’s diversity index (DI) and within group mean genetic distance (GD) in landraces (FIGS Drought and Australia Hot), synthetic hexaploids and elite lines.

LG	FD	AH	SH	E
1A	0.242	0.242	0.224	0.313
1B	0.285	0.273	0.296	0.265
1D	0.252	0.262	0.335	0.264
2A	0.233	0.244	0.256	0.255
2B	0.244	0.265	0.226	0.293
2D	0.216	0.194	0.317	0.226
3A	0.234	0.253	0.237	0.276
3B	0.245	0.276	0.225	0.286
3D	0.186	0.186	0.397	0.196
4A	0.284	0.263	0.242	0.284
4B	0.222	0.211	0.274	0.253
4D	0.203	0.182	0.394	0.243
5A	0.236	0.267	0.204	0.288
5B	0.272	0.261	0.231	0.304
5D	0.182	0.225	0.326	0.203
6A	0.262	0.284	0.251	0.295
6B	0.282	0.282	0.211	0.304
6D	0.283	0.283	0.397	0.315
7A	0.222	0.234	0.245	0.268
7B	0.263	0.305	0.264	0.313
7D	0.224	0.212	0.406	0.172
Mean DI	0.242	0.248	0.284	0.267
Mean GD	0.094	0.105	0.181	0.125

LG: Linkage group; FD = FIGS Drought; AH = Australia Hot; SH = Synthetic hexaploids; E = Elite

### Gene-specific marker diversity in different germplasm sets

The allele frequency for 39 investigated genes (S3 Table) was highly variable in the germplasm groups (Table 2). The gene for grain protein content (*GPC)*, photoperiod insensitivity and vernalization gene alleles *PpdA1a* and *VrnB1b*, and the 1RS:1BL translocations were absent in landraces. *GPC* and *PpdA1a* were present in synthetic hexaploids and *VrnB1b* and 1RS:1BL translocations in elite lines, albeit with low frequencies (Table 2). The vernalization gene allele *VrnA1c* and all seven investigated alleles of the powdery mildew resistance gene (*Pm3*) were absent in the tested elites. The *VrnA1c* allele, which has rarely been found in other wheat collections [17, 18] was found to be present in landraces from Afghanistan, India, Iran and Pakistan (S4 Table). The four powdery mildew resistance alleles (*Pmb*, *Pmc*, *Pmf* and *Pmg*) were present in landraces with frequencies ranging from 0.022 to 0.419 (Table 2). Although with low frequency, two *Pm* (*Pm3f* and *Pm3g)* alleles were also present in SH. The whole collection was devoid of three *Pm* alleles (*Pm3a*, *Pm3d* and *Pm3e*), and the stem rust gene *Sr36* and fusarium head blight gene *Fhb1*. Mean DI based on 39 gene-based markers revealed elite germplasm (DI = 0.15) to be less diverse than FD (DI = 0.16) and AH (DI = 0.17) but more diverse than SH (DI = 0.13).

**Table 2:** Allele frequencies of genes across landraces, synthetic hexaploids and elite lines.

Gene allele	Allele frequency		
	Landraces	Synthetic hexaploids	Elite lines
*RhtB1b*	0.000	0.298	0.839
*RhtD1b*	0.000	0.010	0.145
*LR34*	0.014	0.039	0.120
*Fhb1*	0.000	0.000	0.000
1RS:1BL	0.000	0.000	0.100
*GluA1*	0.027	0.075	0.237
*GluD1*	0.043	0.030	0.745
*Sr36*	0.000	0.000	0.000
*Sbm1*	0.914	0.610	0.929
*GPC*	0.000	0.078	0.000
*PinbD1b*	0.037	0.037	0.019
*PinaD1b*	0.064	0.060	0.925
*PpdD1a*	0.005	0.064	0.944
*PpdA1a*	0.000	0.041	0.000
*Psy 1 D1* ^*+*^	0.011	0.013	0.137
*Psy 1 D1* ^*++*^	0.022	0.006	0.017
*VrnA1a*	0.057	0.005	0.166
*VrnA1b*	0.019	0.086	0.017
*VrnA1c*	0.130	0.000	0.000
*VrnB1a*	0.157	0.063	0.850
*VrnB1b*	0.000	0.000	0.017
*VrnD1*	0.964	0.489	0.900
*GluA3f*	0.016	0.017	0.017
*GluA3b*	0.859	0.894	1.000
*GluA3g*	0.299	0.528	0.083
*GluA3ac*	0.682	0.020	0.933
*GluB3b*	0.561	0.012	0.850
*GluB3i*	0.349	0.017	0.150
*GluB3d*	0.174	0.557	0.133
*GluB3bef*	0.356	0.066	0.450
*GluB3fg*	0.236	0.093	0.216
*Pm3a*	0.000	0.000	0.000
*Pm3b*	0.022	0.000	0.000
*Pm3c*	0.037	0.000	0.000
*Pm3d*	0.000	0.000	0.000
*Pm3e*	0.000	0.000	0.000
*Pm3f*	0.022	0.074	0.000
*Pm3g*	0.419	0.006	0.000
*VpB1*	0.245	0.930	0.931
*PPO33*	0.229	0.039	0.145

*Psy 1 D1+* = Allele for high yellow pigment;

*Psy 1 D1++* = Allele for low yellow pigment

### GBS and gene-based marker diversities in landraces from different geographic regions

**Fig 2:**
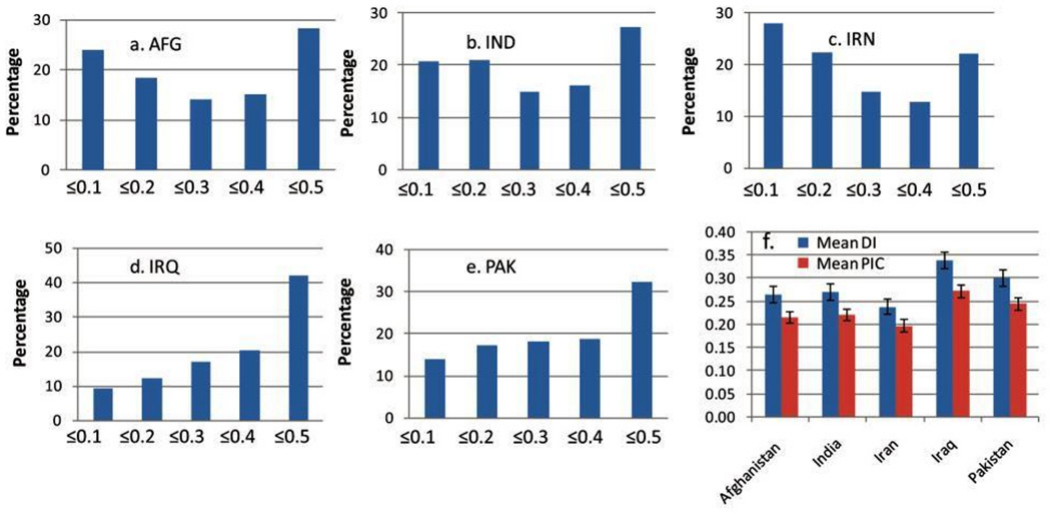
Nei’s diversity index (DI) distribution across Afghanistan (a), India (b), Iran (c), Iraq (d) and Pakistan (e). Each column in *a-e* represents percentage of markers having DI either equal to or less than the value shown on X-axis. Part *f* of figure represents mean DI and PIC across all countries.

The distribution of DIs for landraces from Afghanistan, India, Iran, Iraq, and Pakistan revealed that the latter two groups had the highest percentage of markers with DI between 0.4 and 0.5 (Fig 2a–2e). Mean DI and polymorphic information content (PIC) values revealed that landraces from Iraq formed the most diverse group followed by those from Pakistan (Fig 2f). Diversity estimates based on gene-based markers also showed the highest diversity in landraces from Iraq (S3 Fig). S4 Table presents the frequency of 39 gene-specific alleles in landraces from five countries.

### Neighbor joining dendrogram

**Fig 3:**
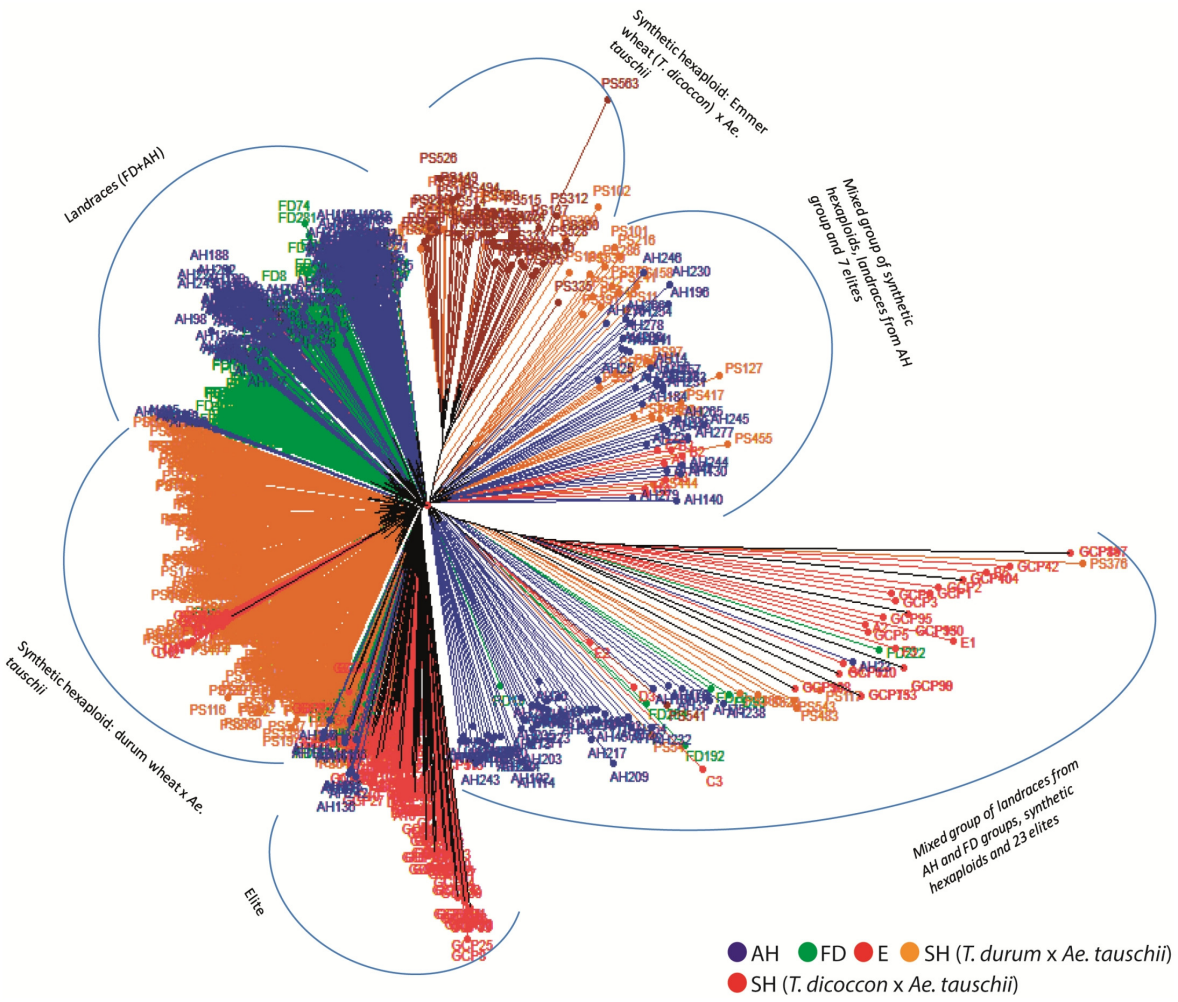
Neighbor joining dendrogram based on GBS markers using Euclidean distance showing genetic relationships among four germplasm sets.

**Fig 4:**
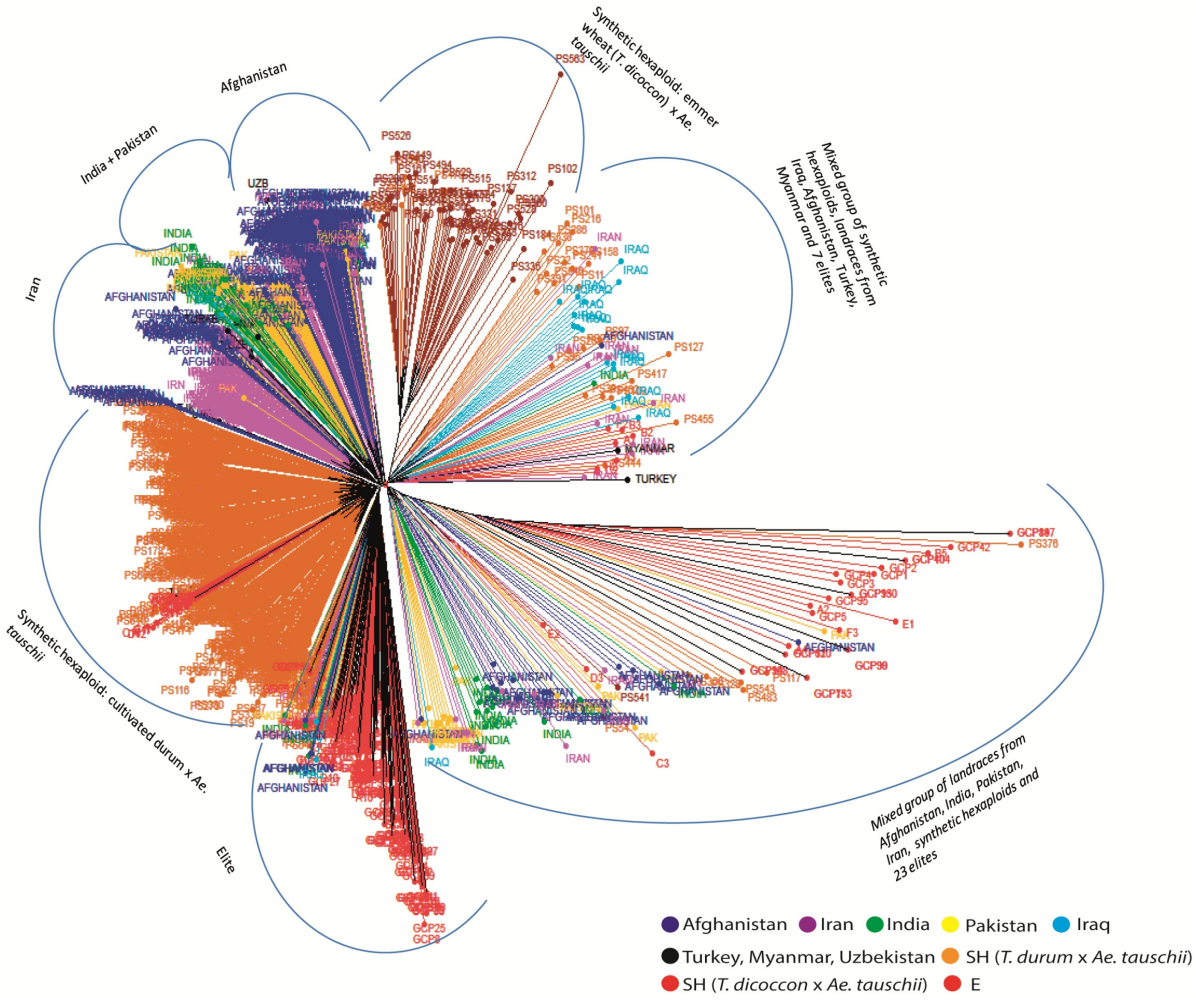
Neighbor joining dendrogram based on GBS markers using Euclidean distance showing genetic relationships of landraces from different geographic origins with elites and synthetic hexaploids.

The neighbor joining (NJ) tree divided the four germplasm sets (FD, AH, SH and E) into six groups (Fig 3). Eighty five percent of landraces from the FD and AH groups formed one group and the remaining 15% of landraces (mainly from the AH group) dispersed in two mixed groups composed of landraces, SH and E. The SH were divided into two groups; one bigger group with 581 SH made by crossing durum wheat (*T*. *turgidum* ssp. *durum*) and *Ae*. *tauschii*, and, the other one with only 47 genotypes made by crossing emmer wheat (*T*. *dicoccon*) and *Ae*. *tauschii*. The remaining SH were dispersed in two mixed groups. The elite germplasm was dispersed in four different groups in the dendrogram. The group labelled as Elite (Fig 3) was the biggest group of elites constituting 163 (77.2%) accessions. The remaining elites were either dispersed in the two mixed groups (14.2%) or were part of the bigger SH group (8.5%). The second NJ tree (Fig 4) shows the geographic origin of landraces. The landraces from Iraq were predominant in one of the mixed groups and those from Afghanistan, India and Pakistan were predominant in the second mixed group. A few landraces were also present in the group that contained the 163 elites (Elite group). In the landrace group, majority of the genotypes from Afghanistan and Iran clustered separately, whereas genotypes from India and Pakistan formed one mixed group. We compared the levels of diversity in the two groups of SH obtained in the dendrograms which revealed non-significant differences between them (S4 Fig).

### Coefficient of gene differentiation and gene flow

Estimates of the coefficient of gene differentiation (G_st_) revealed that there was more divergence between the elite germplasm and landraces than between landraces of different geographic origins (Table 3). These results were confirmed by gene flow (N_m_) analyses. Gene flow between elite germplasm and landraces was lower than among landraces of different origins (Table 3).

**Table 3:** Apportionment of genetic variation between different germplasm sets.

	H_t_	H_s_	G_st_	N_m_
Elite lines and landraces	0.360	0.301	0.165	2.547
Landraces from five different countries*	0.299	0.264	0.118	3.755

* Afghanistan, India, Iran, Iraq and Pakistan

Ht = Total genetic diversity

Hs = Within population diversity

Gst = Coefficient of gene differentiation

Nm = Gene flow

### Novel alleles for known genes of agronomic importance

Screening of germplasm sets with the known allele-specific markers for vernalization and glutenin genes (S3 Table) revealed novel bands for *Vrn-A1c*, *vrn-B3*, *GluA3b*, *GluB3g* and *GluB3i* in the landraces of different origins. Screening of landraces for the *Vrn-A1c* allele revealed an expected band of 1170 bp in 13% of landraces. However, in some landraces originating from Iraq (9), Iran (4) and Afghanistan (2), a band of ~ 600 bp was obtained, thus indicating a deletion in the *Vrn-A1c* allele (Fig a in S5 Fig). Similarly, a band of ~ 1900–2000 bp was observed in the landraces from Pakistan (2), Iran (7), Uzbekistan (1), Turkmenistan (1) and Afghanistan (2) instead of an expected band size of 1140 bp for allele *vrnB3*, indicating an insertion of approximately 800–900 bp (Fig-b in S5 Fig). Novel alleles for *GluA3b*, *GluB3g* and *GluB3i* were also identified with greater band sizes than expected (bands of 894, 853 and 621 bp, respectively) in 19 landraces originating from Pakistan, Afghanistan, India and Turkmenistan (Figs a, b and c in S6 Fig).

### Sequence variation analysis of novel alleles

**Fig 5:**
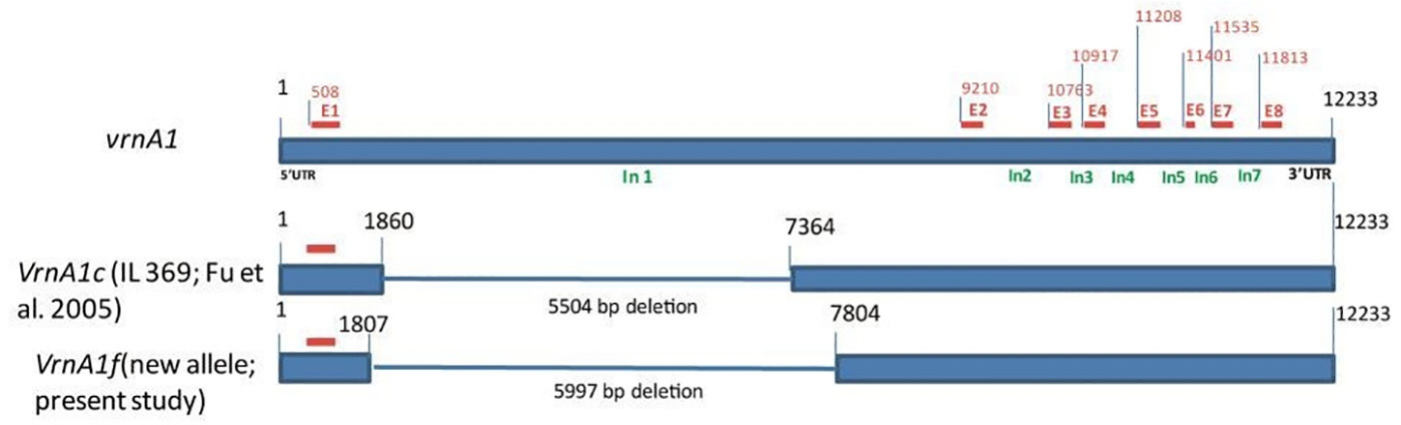
Schematic diagram of *vrn-A1 gene*, *Vrn-A1c* and new allelic variant *Vrn-A1f*. The eight exons are show as red boxes with numbers E1 to E8 and introns are shown as In1 to In 7. The novel deletion is shown in intron 1 between 1807 and 7804 bp relative to recessive allele *vrn-A1* (AY747600) and dominant allele *Vrn-A1c* (AY747599). Nucleotide numbers are based on the sequence of *vrn-A1* (AY747600).

The new allelic bands of *Vrn* genes were cloned, sequenced and aligned with known *vrn-A1*, *Vrn-A1c* and *vrn-B3* alleles (17, 18). The sequence alignment of 600 bp band with recessive winter allele *vrn-A1* (AY747600) and *Vrn-A1c* (AY747599) revealed a novel deletion of 5997 bp in intron 1 of winter allele *vrn-A1*. This novel deletion represents an additional deletion of 493 bp in intron 1 of winter allele *vrn-A1*, along with a 5504 bp deletion in *Vrn-A1c* (Fig 5). The new *Vrn-A1* allele is named as *Vrn-A1f* and submitted to NCBI GenBank (Accession no. KR824429). Similarly, the alignment of the new *Vrn-B3* allele with the recessive *vrn-B3* allele revealed a large insertion of 890 bp into the 5′untranslated region (UTR) of *vrn-B3*, and an additional deletion of 1 bp and three SNPs outside this large insertion (S7 Fig). Sequence analysis of new *Glu* alleles is underway.

## Discussion

As humanity confronts the nexus of ever-rising food demands and climate change, the need to exploit the full potential of wheat genetic resources to accelerate performance gains has become more urgent. Wheat genetic resources from gene banks need to be characterized to channelize useful genetic variation into modern elite gene pools. This is the first report of genetic characterization of a very large (1,423 accessions) set of wheat germplasm using GBS and gene-specific markers. Recently, Manickavelu et al. [19] reported diversity of 446 Afghan wheat landraces with GBS markers. The GBS technology has the potential to provide an in-depth and a robust diversity estimate with much reduced ascertainment bias as compared to other whole-genome-genotyping technologies [20]. It can also unveil new and favorable genetic variations in gene bank accessions, thus enabling a targeted choice of accessions with high value for pre-breeding [21]. A number of genetic diversity studies have been conducted for wheat using marker systems other than GBS [10, 22–35].

The landrace and elite materials investigated in the study represented major spring wheat growing environments of the world. Particularly, the landraces were collected from drought and heat prone environments using a specific trait-based FIGS approach adopted by ICARDA gene bank. It is a highly innovative approach based on the assumption that drought- and themostress- tolerant landraces are prevalent in areas where stress has been most severe—the phenomenon of co-evolution [36]. The accessions originating from such regions are collected and pursued further and only the highest potential accessions are then confirmed in field experiments. Using this approach novel sources of resistance in wheat to drought, heat, salinity and to several diseases and insect pests have been successfully identified [37].

We identified thousands of new SNP variations specific to drought and heat tolerant accessions (S1 Fig). Some of these SNP variations can be incorporated to elite genotypes after investigating their allelic effects with genome wide association analysis (GWAS). The novel superior alleles thus identified in GWAS can be fitted into genomic prediction models to realize genetic gains through genomic selection. This approach is currently being followed in the SeeD-Wheat project at CIMMYT. This has opened new avenues for enriching the elite germplasm with novel drought and heat tolerant genes and for further broadening the diversity of elite germplasm. Among landrace accessions, those from Iraq were the most diverse followed by Pakistan (Fig 2). The higher diversity in Iraq and Pakistan, even greater than Iran, is unexpected from known evolutionary history of bread wheat as Iran is one of the main centers of evolution of wheat. However, it should be noted that only drought and heat tolerant genotypes were collected from Iran in the FD and AH sets, respectively. Thus, they are not representative of entire geographical diversity in Iran. Although this trait-based selection has limited the overall diversity in the tested landraces (Table 1), it has provided useful alleles and resources to breed for heat and drought prone environments.

In the NJ dendrogram, 77% of the elite lines and 85% of the landraces formed separate clusters; 96% SH also grouped into two clusters separate from elites (Figs 3 and 4). Further, high genetic differentiation (Gst) between elites and landraces was observed (Table 3). These results explicitly indicate a) the divergence of the tested elites from the landraces and SH and b) landraces and SH as two different pools of genetic variation for further broadening the genetic base of elite germplasm. Two groups were evident in SH which were divided according to the tetraploid parent used in the crosses; durum wheat x *Ae*. *tauschii* and emmer wheat x *Ae*. *tauschii*. In the CIMMYT wide crossing program most synthetics were produced using modern durum wheats (*T*. *turgidum* subsp. *durum*), while only a few dozen combinations included emmer wheat (*T*. *dicoccon*) [38]. Diversity estimates in two groups of SH did not differ significantly (S4 Fig), which indicates that both tetraploid parents have contributed equally to the diversity of SH. The diversity information of landraces and SH from two different origins (landraces: FD and AH; SH: *T*. *turgidum-* and *T*. *dicoccon-*based) has been integrated into the wheat breeding pipelines to introgress novel variations into high yielding and widely adapted elite backgrounds. More than 200 diverse accessions have been identified for pre-breeding and for allele mining of candidate genes for drought and heat tolerance.

The higher number of GBS SNP markers specific to SH than landraces (S1 Fig) was not unexpected considering that such gain of novel DNA fragments is common after polyploid formation [39]. Several mechanisms such as homoeologous recombination, point mutation, transposon activation and gene conversion-like events have been reported to generate novel genetic changes in polyploids [39]. From a breeder’s perspective, these results are significant as some of this variation may provide novel alleles to wheat breeders for traits not yet tapped in the primary gene pool of wheat. Dreisigacker et al. [11] also reported several novel bands in SH with SSR markers which were stably inherited in synthetics-derived backcrossed lines. Thus, a detailed scrutiny of the novel GBS SNP tags in SH is required to identify the genes worth introgressing into elite germplasm.

Comparison of the diversity of the tested elite germplasm vis-á-vis previous reports (S5 Table) with SNP markers on elite germplasm of other breeding programs revealed that the elite lines of the present study are more diverse than most other breeding programs [40–44]. This result supports previous conclusions that CIMMYT breeders successfully broadened the genetic diversity of the elite germplasm through incorporation of primary synthetics into the breeding programs [10, 11, 45] and also via consistent introductions of exotic materials from all over the world [10, 46, 47].

The diversity pattern obtained from 39 allele-specific markers for different adaptive and quality traits’ genes showed an order: landraces>elites>SH. This order is opposite to what was observed with GBS-based diversity (Table 1). These results were expected as most of the adaptive and quality genes have been fixed in the elite germplasm through years of breeding (Table 2). Genic diversity analysis further demonstrated that landraces from Iraq are the most diverse (S3 Fig), which is in accordance with the GBS marker-based results. The most significant output of assessing genic diversity was the identification of novel alleles for various agronomically important genes (Fig 5, S5 and S6 Figs). Two allelic variations in *Vrn-A1* and *Vrn-B3* genes, associated with deletions and insertions, respectively, were identified. The sequence differences in the promoter region and large insertions or deletions in the intron I of the *Vrn-1* locus have been reported to be associated with spring vs. winter growth habit [17, 18]. Allele *Vrn-A1c* carries a large 5504 bp deletion in intron I of recessive allele *vrn-A1* [17]. We detected a novel deletion of 5997 bp, named *Vrn-A1f*, in the intron I region of recessive gene *vrn-A1* which extended 440 bp further downstream and 53 bp upstream from the deletion in *Vrn-A1c* (Fig 5). *Vrn-A1f* was observed in landraces from Iran, Iraq and Afghanistan, thus pointing to a Middle East and/or near eastern origin of this allele. Similarly, we detected an insertion of 890 bp in the 5’ UTR region of promotor of *vrn-B3*, and an additional 1-bp deletion and three SNPs outside this large insertion in the promotor region. Derakhshan et al. [48] reported a similar size insertion in the *vrn-B3* gene from Iranian landraces. The authors, however, did not sequence the band. Chen et al. [49] cloned the 2000 bp band and reported a 890 bp insertion in a Chinese cultivar Chadianhong, and named it as *Vrn-B3b*. Comparison of the sequences of insertions in Chadianhong and the landraces of this study revealed an identical 890 bp insertion (S7 Fig), thus indicating the presence of *Vrn-B3b* allele in landraces of the present study. *Vrn-B3b* was identified in the landraces from Pakistan, Iran, Uzbekistan, Turkmenistan and Afghanistan, thus indicating a wide distribution of this allele. Preliminary evidence suggests that *Vrn-A1f* promotes flowering by six to seven days (Fig a in S8 Fig), and *Vrn-B3b* delays flowering by ten days (Fig b in S8 Fig) as also reported in Chadianhong [49].

Heading time is a major determinant of wheat’s adaptation to different environments, and critical in minimizing the risk of frost, heat, and drought for reproductive development. In future climate change scenarios, the interplay of *Ppd* and *Vrn* genes will have important implications for improving yield by controlling flowering time [50]. An in-depth crop modelling simulation study taking into account 35 possible climate scenarios revealed that photoperiod-sensitive cultivars of millet and sorghum are more resilient to future climate conditions than modern photoperiod-insensitive cultivars [50]. In this regard, *Vrn-A1f* and *Vrn-B3b* alleles identified in photoperiod-sensitive landraces adapted to heat and drought prone environments could be very efficiently utilized for developing climate smart wheat varieties. The effects of *Vrn-B3b* allele on yield have not been yet investigated (49). Responses of *Vrn-A1f* and *Vrn-B3b* alleles on grain yield are currently under investigation for their efficient utilization in the wheat breeding. We are also analyzing the interactions of the above said alleles with previously reported ones to determine a suitable combination for introgression into elite wheat genotypes. New alleles of *Glu* genes were also observed in landraces from Pakistan, Iran, Turkmenistan, India and Afghanistan (Figs a, b and c in S6 Fig). Allelic variations at the *Glu-3* loci (encoding low molecular weight glutenin subunits) have a pronounced effect on the visco-elastic properties of wheat dough [51]. The effect of these novel variations on visco-elastic properties is also under investigation, particularly the novel *Glu-B3g* allele (Fig-a in S6 Fig), as positive effect of *Glu-B3g* on peak mixing time (a parameter of strong dough) has already been established [52].

The agronomically important alleles controlling highly heritable traits such as heading, height and pre-harvest sprouting (*Ppd-D1a*, *Vrn-B1a*, *Vrn-D1*, *Rht-B1b*, *Vp-B1*) were almost fixed in the tested elite germplasm (Table 2). Of the various diseases of wheat, resistance to soil borne mosaic virus is highly heritable being controlled by a single locus, *Sbm1* [53]. This gene was also fixed in the elite lines (Table 2). The genes/gene alleles controlling less heritable traits (resistance to leaf rust, powdery mildew, fusarium head blight) were present either in moderate frequency (*LR34*) or were absent (seven *Pm* alleles, *Fhb1*) in the tested elite lines. It is noteworthy, however, that elite germplasm display significant resistance for fusarium head blight and powdery mildew, which could have resulted from selection of yet unknown or uninvestigated genes/alleles. Among the quality traits, grain hardness is extremely important and forms the basis of differentiating within the world trade of wheat grain. The trait is related to the variation in two puroindolines (Pin A and Pin B) encoded by *Pina* and *Pinb* genes, respectively. The absence or mutation of either of these genes results in hard texture (54). The tested elite lines showed almost fixed *Pina* gene (*Pina-D1b* frequency 92.5%), whereas the frequency of the *Pinb* gene (*Pinb-D1b*) was only 1.9% (Table 2). Previous studies have reported significant advantage of *Pinb-D1b* allele over *Pina-D1b* for milling and bread quality traits [54]. The *Pinb-D1b* allele was identified in 16 landraces from diverse origins (S4 Table) and 4 SH in this study which can be introgressed into elite germplasm to increase the allelic variability of this locus. This study has confirmed the potential benefits related to the use of landraces and synthetic wheats as exotic parents to introduce new allelic diversity into breeding programs. Germplasms resources are freely available for the global wheat community.

## Conclusions

The results of this study suggest that there is significant unexploited variation in landraces and SH that can be channeled into modern cultivars. This genetic variation, when combined with existing genetic variation in the elite wheat gene pool, will further improve stress adaptation and quality traits and also enrich it with novel drought and heat tolerance genes. Efforts are being made to maximize variation for heat and drought tolerance alleles in elite genotypes to complement wheat improvement activities. Based on the marker information generated in this study, more than two hundred landraces and synthetic hexaploids are being used for pre-breeding and generating bridging germplasm. An ‘allele-mining panel’ has also been assembled for allele mining of candidate genes for drought and heat stress tolerance. The new allelic variation identified for vernalization and glutenin genes will be incorporated into breeding program once their effects on yield and quality parameters are validated. The lines carrying the new alleles can be made available to the researchers worldwide on request.

## Materials and Methods

### Plant material

A total of 1,423 wheat germplasm accessions were characterized in this study (S1 Table). These included 561 landrace accessions representing three geographic regions (Near East, Middle East and South West Asia), 651 synthetic hexaploids developed at CIMMYT by crossing durum wheat (*T*. *turgidum* subsp. *durum*) or emmer wheat (*T*. *dicoccon*) with diverse *Aegilops tauschii* accessions, as well as 211 cultivars and elite lines. Of the 561 landrace accessions, 280 landraces were obtained from ICARDA. These landraces were identified as drought tolerant using a focused identification of germplasm strategy (FIGS) approach (http://www.icarda.org/tools/figs) and were denoted as ‘FIGS Drought’ (FD) in this study. Remaining 281 landrace accessions were obtained from Australian gene bank, Horsham, Victoria. These landraces were identified as heat tolerant. This set was denoted as ‘Australia Hot’ (AH).

### Genotypic characterization

Genomic DNA was extracted from fresh leaves collected from a single individual plant per accession using a modified CTAB (cetyltrimethylammonium bromide) method [55] and quantified using NanoDrop 8000 spectrophotometer V 2.1.0. For genotypic characterization, a next-generation sequencing technique called DArTseq was employed. A complexity reduction method including two enzymes was used to generate a genome representation of the set of samples. *PstI*-RE site specific adapter was tagged with 96 different barcodes enabling multiplexing a plate of DNA samples to run within a single lane on Illumina HiSeq2500 instrument (Illumina Inc., San Diego, CA). The successful amplified fragments were sequenced up to 77 bases, generating approximately 500,000 unique reads per sample. Thereafter the FASTQ files (full reads of 77bp) were quality filtered using a Phred quality score of 30, which represent a 90% of base call accuracy for at least 50% of the bases. More stringent filtering was also performed on barcode sequences using a Phred quality score of 10, which represent 99.9% of base call accuracy for at least 75% of the bases. A proprietary analytical pipeline developed by DArT P/L was used to generate allele calls for SNP and presence/absence variation (PAV) markers. Then, a set of filtering parameter was applied to select high quality markers for this specific study. One of the most important parameters is the average reproducibility of markers in technical replicates for a subset of samples, which in this specific study was set at 99.5%. Another critical quality parameter is call rate. This is the percentage of targets that could be scored as '0' or '1', the threshold was set at 50%. PAV’s markers were not used in this study.

### Gene-based marker genotyping

Sequence tagged site (STS) markers reported on the MASWheat (http://maswheat.ucdavis.edu/protocols/index.htm) database for various agronomic traits, as well as for quality and disease resistance genes were used for genotyping using PCR protocols and gel electrophoresis procedures described in this database. In addition, genotyping was done using SNP markers designed from wheat gene sequences, reported on CerealsDB (http://www.cerealsdb.uk.net/cerealgenomics/CerealsDB/kasp_download.php?URL=), using the KASPar genotyping system (KBiosciences, UK). The allele-specific gene-based STS and SNP markers used in this study are listed in S3 Table and their primer sequences are described on MASWheat and CerealsDB databases.

### Cloning, nucleotide sequencing and analysis

The novel bands were cloned and sequenced using the commercial service provided by the Molecular Biology Service Centre, Simon Fraser University, Vancouver, BC, Canada. A standard T/A cloning procedure using pGEM-T Easy vector (Promega) was used [56]. Sequencing chromatograms were analyzed using Chromas Version 1.4.5. Sequencing data of novel vernalization-gene fragments were aligned with sequences of *vrn-A1* (AY747600), *Vrn-A1c* (AY747599) and *vrn-B3* (DQ890162) genes using the BLAST2 sequences option of BLASTN program available at NCBI (http://www.ncbi.nlm.nih.gov/), database and the CLUSTAL X programme [57]. A default setting with a fixed gap penalty of 6.66, and a 0.5 DNA transition weight in the multiple alignment parameter option was opted for alignment.

### Diversity analysis

The map positions of GBS SNP markers were obtained from a 64K consensus map provided by DArT Pvt. Ltd., Australia. The number of mapped markers for the A, B and D genomes was 3964 (33.3%), 4294 (36.1%) and 3616 (30.4%), respectively. Before diversity analysis, markers were filtered using the criterion; missing data < 20% and minor allele frequency > 0.05.

Two diversity parameters, Nei’s diversity index (DI) and polymorphic information content (PIC), were calculated to characterize the genetic diversity of A, B and D genome-based GBS markers, gene-based markers, and of different germplasm sets using the “Genetics” package in R (http://www.r-project.org/) and POPGENE version 1.32 [58]. To compare the diversity of landraces from different geographic origins, countries with minimum 20 representatives (Iran, Iraq, India, Pakistan and Afghanistan) were included in analysis.

Nei’s genetic diversity statistics [59] was used to measure total genetic diversity (H_t_) as well as intra-population (H_s_) genetic diversity. The coefficient of gene differentiation (G_ST_) was calculated as G_ST_ = 1—H_s_ / H_t_. Gene flow was estimated as Nm = 0.5 x (1—Gst)/Gst. Genetic relationships were inferred by obtaining a distance matrix (using Euclidean distance) with GBS SNP markers using a custom R function and then using the distance matrix for constructing a neighbor joining dendrogram. The confidence interval of the genetic relationships among the accessions was determined by performing 1000 bootstraps. The genetic groupings were confirmed using DARwin v 5.0.158 [60].

## Supporting Information

S1 Fig GBS marker distribution in different germplasm sets. The underlined number represent markers specific to each group and markers alongside arrows represent those shared exclusively between two groups. Markers shared among any three and all four groups are not shown. FD; FIGS Drought, AH; Australia Hot, SH; Synthetic Hexaploids, E; Elite germplasm.
(TIFF)

S2 Fig Comparison of diversities of A, B and D sub-genomes across FIGS Drought (FD), Australia Hot (AH), synthetic hexaploids (SH) and elite (E) using all samples (Fig a), using 211 samples in each group (Fig b) and DI_AB_: DI_D_ in landraces (FD+AH), SH and E (Fig c). (TIFF)

S3 Fig Mean DI (blue) and PIC (red) across all countries based on gene-based markers. (TIFF)

S4 Fig Mean DI and PIC in two sets of synthetic hexaploids. (TIFF)

S5 Fig Polymerase chain amplification with *Vrn-A1c* (Fig a) and *vrn-B3* (Fig b) allele specific primers in landraces. The amplified bands in three (Fig a) and two landraces (Fig b) are smaller and larger, respectively, than the expected sizes (1170 and 1140 bp for *Vrn-A1c* and *vrn-B3*, respectively).
(TIFF)

S6 Fig Polymerase chain amplification with *GluB3g* (Fig a), *GluA3b* (Fig b) and *GluB3i* (Fig c) allele specific primers in landraces. The amplified bands in a few landraces are larger than the expected sizes (853, 894 and 621bp for *GluB3g*, *GluA3b* and *GluB3i*, respectively).
(TIFF)

S7 Fig Alignment of *Vrn-B3* vernalization allele identified in landraces of present study (*Vrn-B3L*) with recessive *vrn-B3* and *Vrn-B3b*. (DOC)

S8 Fig Phenotypic effects of novel alleles *Vrn-A1f* (Fig a) and *Vrn-B3b* (Fig b) on flowering time. The landrace accession carrying *Vrn-A1f* deletion (Fig a; left pot) flowers six to seven days earlier than the line without this deletion (Fig a; right pot). The landrace accession carrying *Vrn-B3b* insertion flowers ten days later (Fig b; left pot) than the line without this insertion (Fig b; right pot).
(TIFF)

S1 Table Landraces, Synthetic hexaploids and elites used in the study. (XLSX)

S2 Table Nei’s diversity index (DI) in landraces (FIGS Drought and Australia Hot), synthetic hexaploids and elite lines using 211 samples in each group. (DOCX)

S3 Table Thirty nine allele specific gene-based SNP and STS markers used in the present study. (DOCX)

S4 Table Allele frequencies of gene alleles in the landrace germplasm from different countries. (DOCX)

S5 Table Review of wheat diversity studies using different marker systems. (XLSX)
