# Supplementary figures and images for: Exploring and Mobilizing the Gene Bank Biodiversity for Wheat Improvement
Source: PLoS One. 2015 Jul 15;10(7):e0132112. doi: 10.1371/journal.pone.0132112 (PMC4503568; doi:10.1371/journal.pone.0132112)

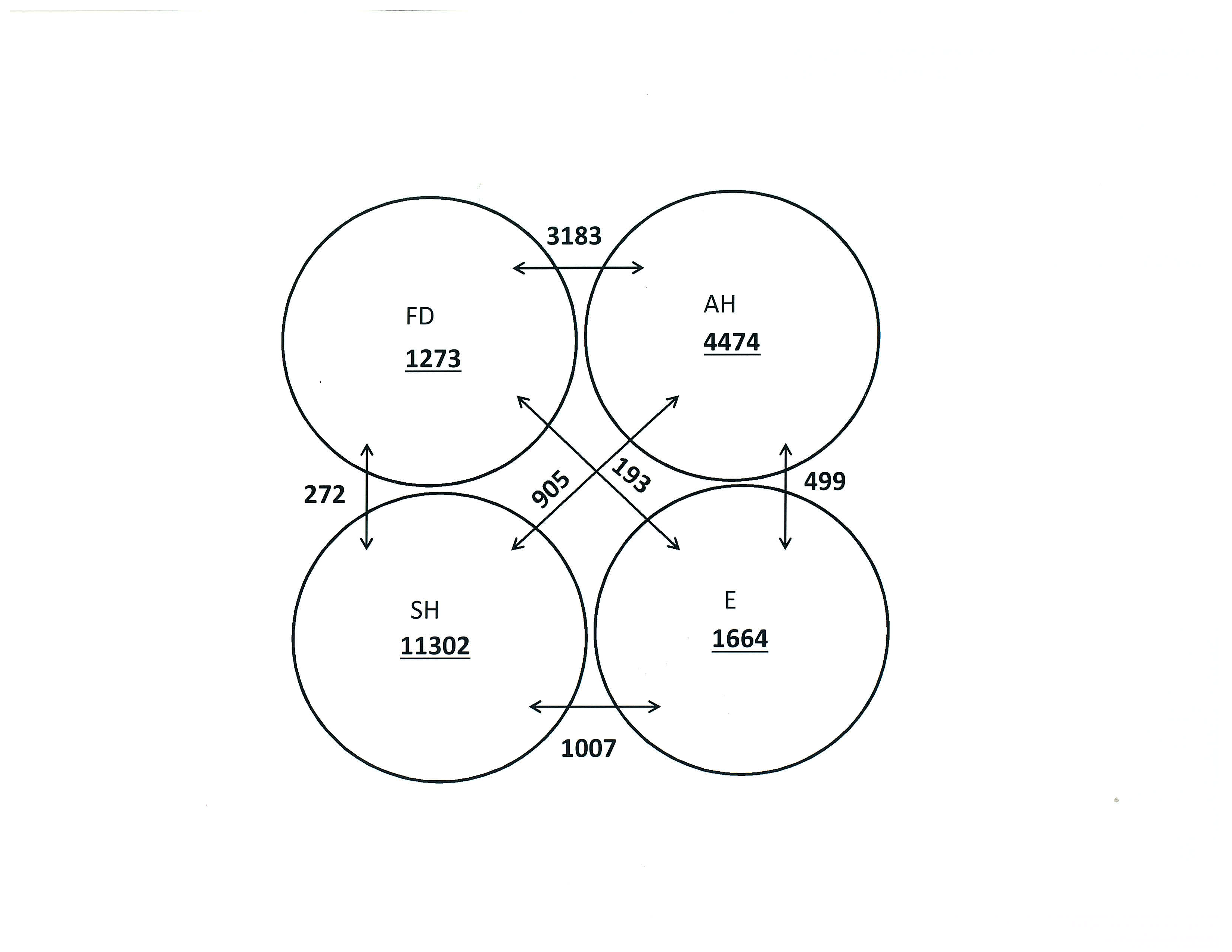

Supplement: S1 Fig — The underlined number represent markers specific to each group and markers alongside arrows represent those shared exclusively between two groups. Markers shared among any three and all four groups are not shown. FD; FIGS Drought, AH; Australia Hot, SH; Synthetic Hexaploids, E; Elite germplasm. (TIFF) [file pone.0132112.s001.tiff]

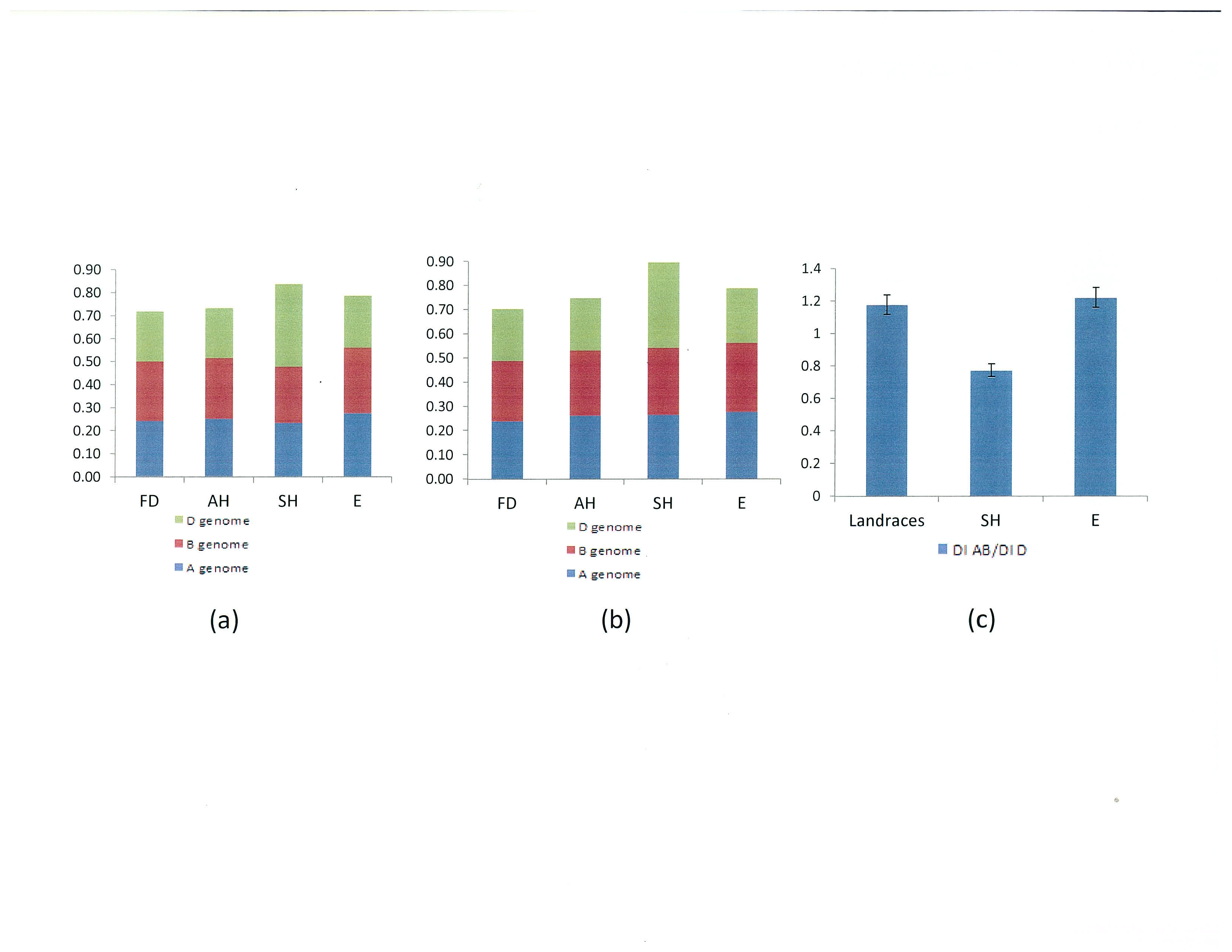

Supplement: S2 Fig — (TIFF) [file pone.0132112.s002.tiff]

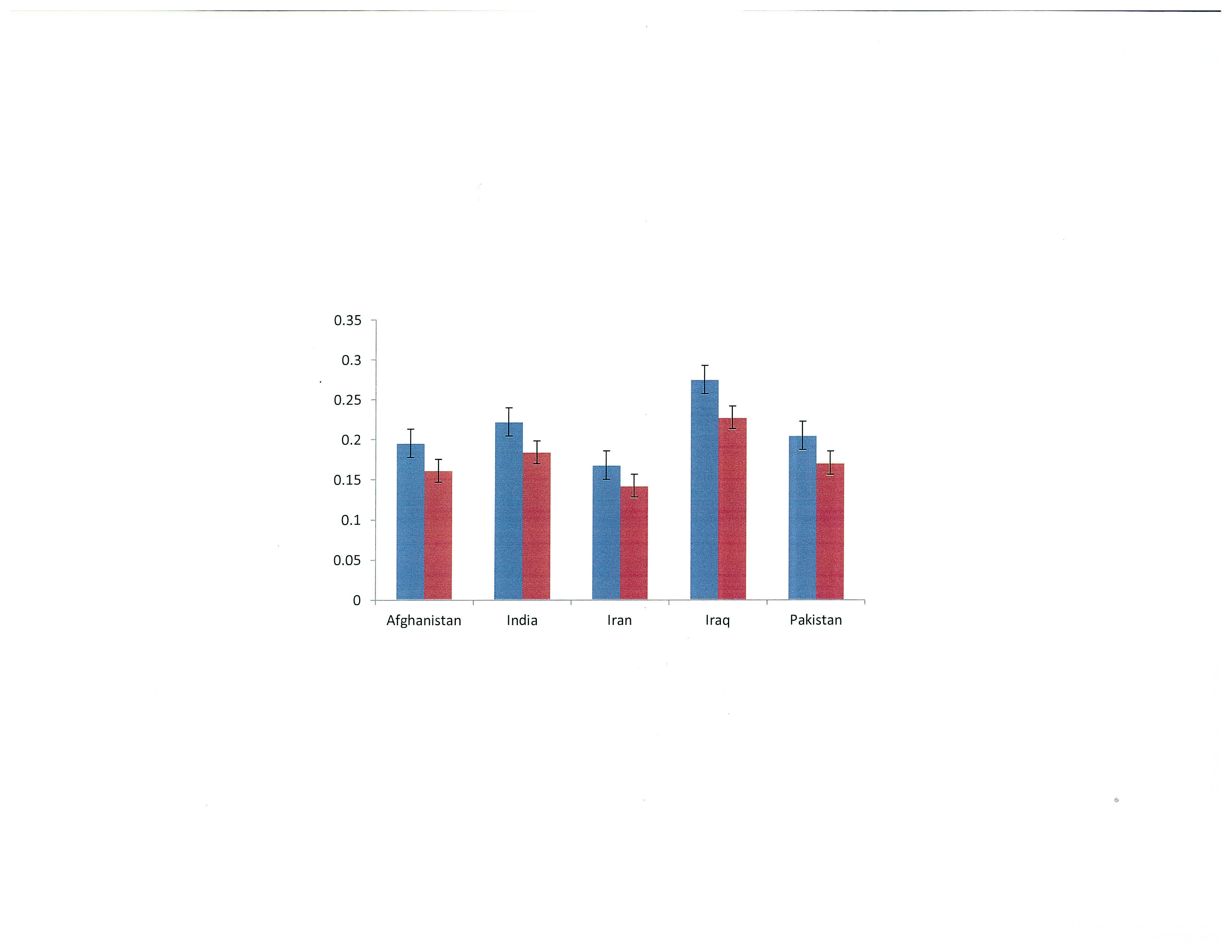

Supplement: S3 Fig — (TIFF) [file pone.0132112.s003.tiff]

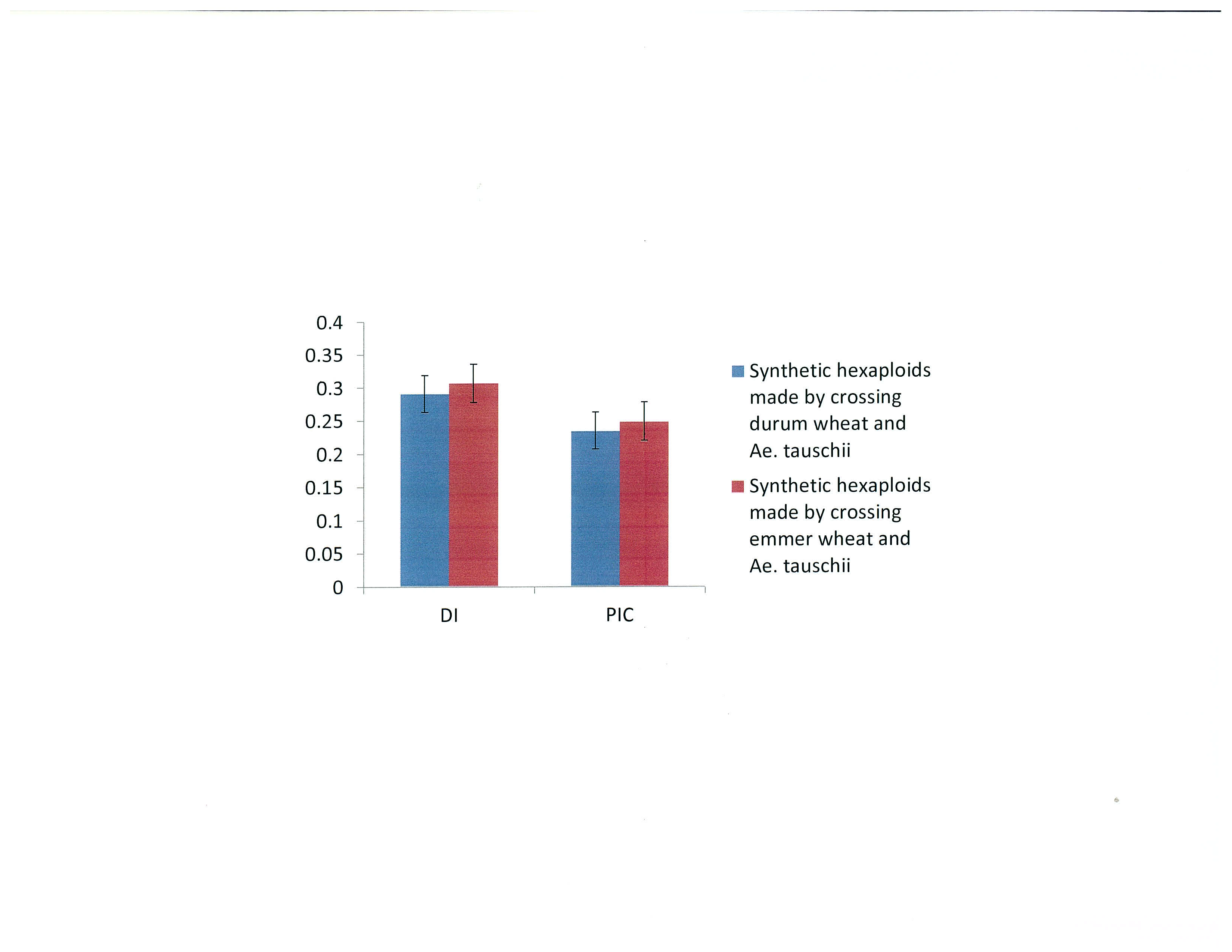

Supplement: S4 Fig — (TIFF) [file pone.0132112.s004.tiff]

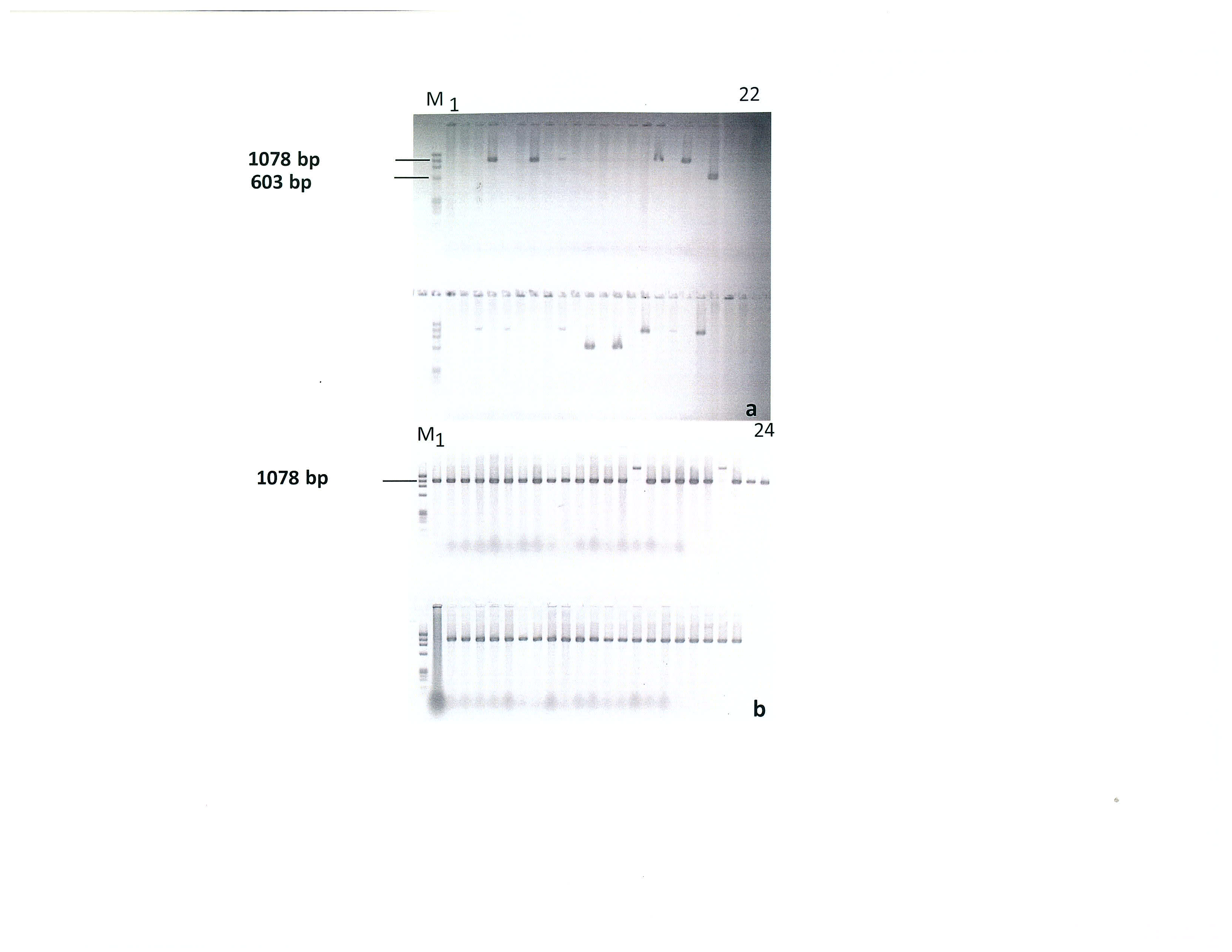

Supplement: S5 Fig — The amplified bands in three (Fig a) and two landraces (Fig b) are smaller and larger, respectively, than the expected sizes (1170 and 1140 bp for Vrn-A1c and vrn-B3, respectively). (TIFF) [file pone.0132112.s005.tiff]

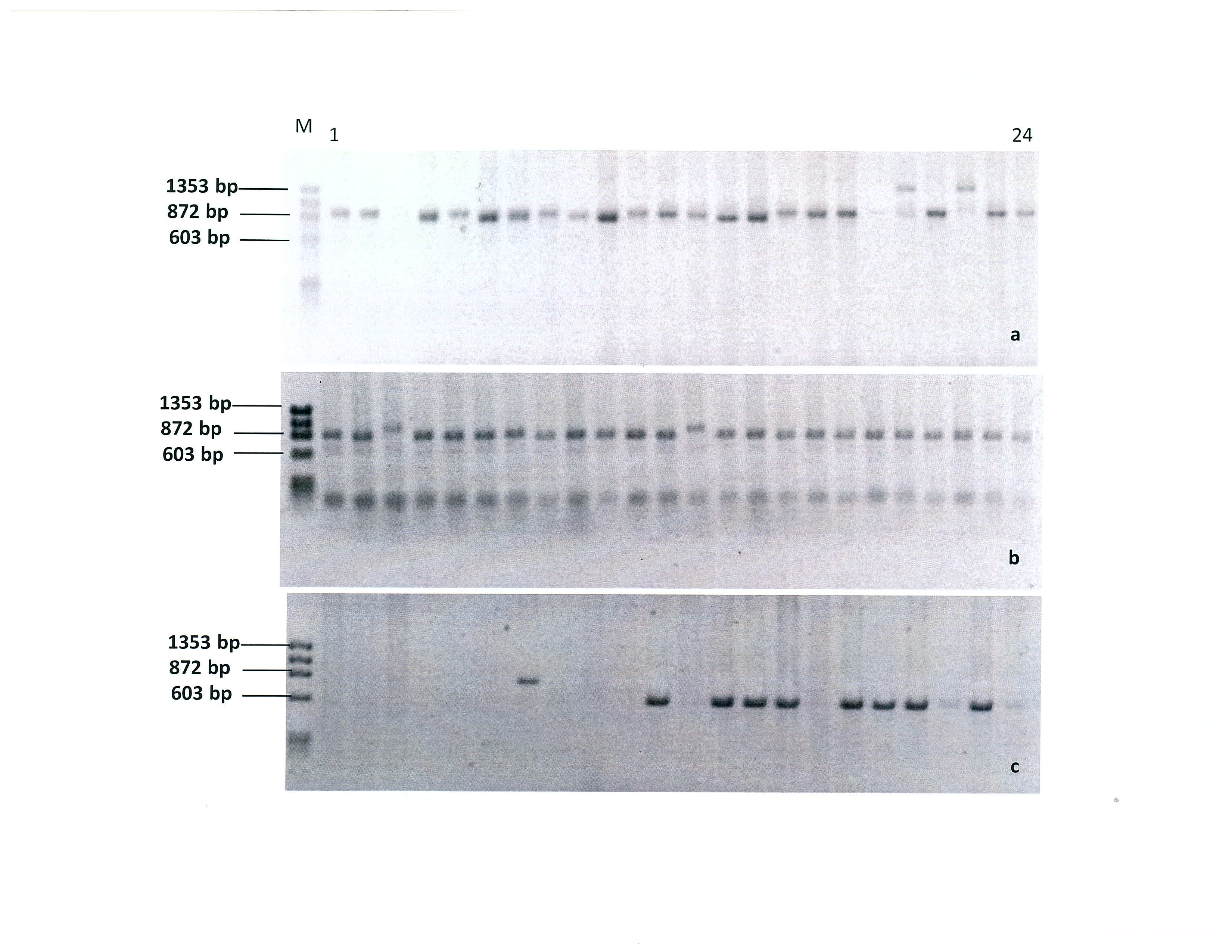

Supplement: S6 Fig — The amplified bands in a few landraces are larger than the expected sizes (853, 894 and 621bp for GluB3g, GluA3b and GluB3i, respectively). (TIFF) [file pone.0132112.s006.tiff]

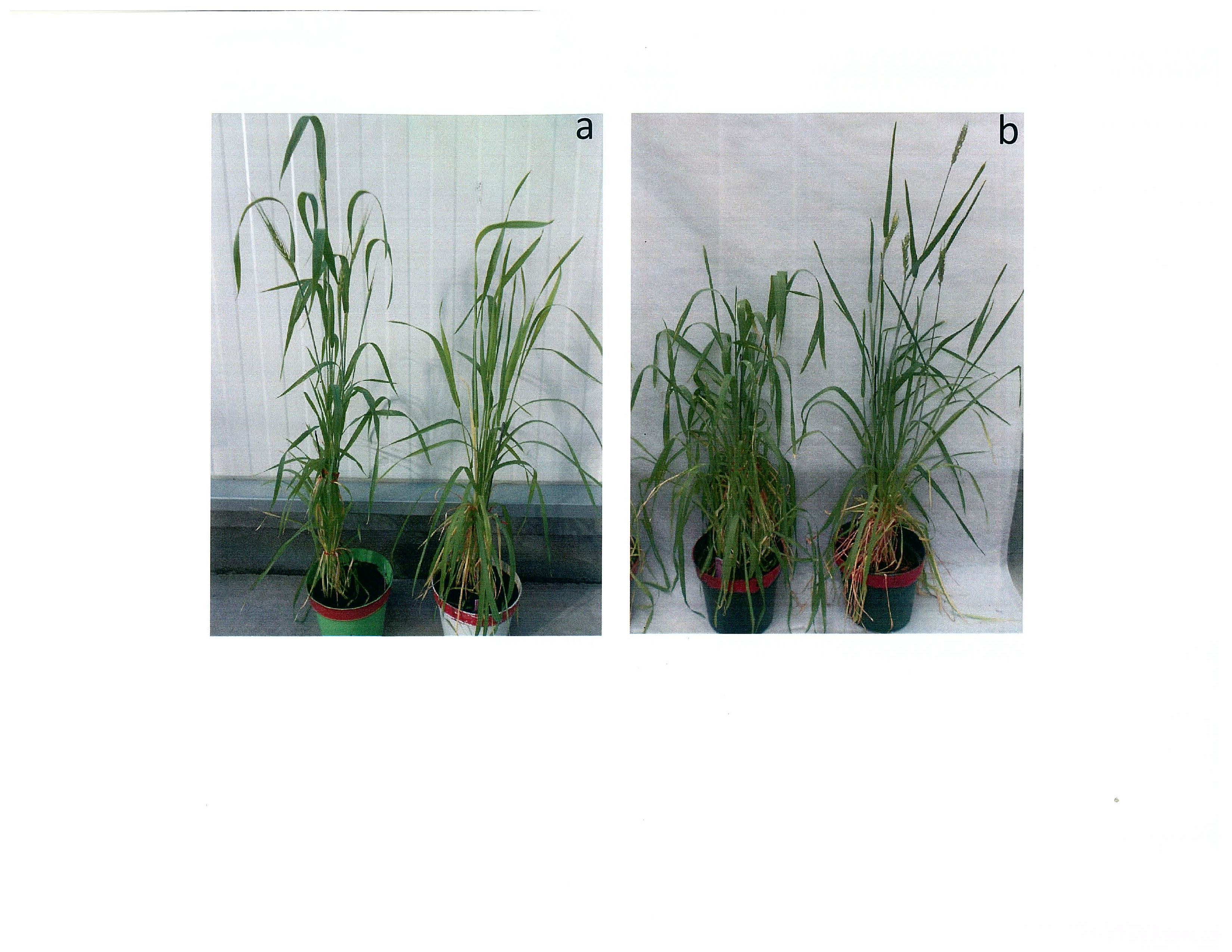

Supplement: S8 Fig — The landrace accession carrying Vrn-A1f deletion (Fig a; left pot) flowers six to seven days earlier than the line without this deletion (Fig a; right pot). The landrace accession carrying Vrn-B3b insertion flowers ten days later (Fig b; left pot) than the line without this insertion (Fig b; right pot). (TIFF) [file pone.0132112.s008.tiff]
