# Supplementary material for: Exploring and Mobilizing the Gene Bank Biodiversity for Wheat Improvement
Source: PLoS One. 2015 Jul 15;10(7):e0132112. doi: 10.1371/journal.pone.0132112 (PMC4503568; doi:10.1371/journal.pone.0132112)
Supplement: S7 Fig — (DOC) [file pone.0132112.s007.doc]

Vrn-B3L ATGCTTTCGCTTGCCATCCCATCTCAGGAAACGTTCGCCAGACAGCATTCCTATTATTAA 60

Vrn-B3b ATGCTTTCGCTTGCCATCCCATCTCAGGAAACGTTCGCCAGACAGCATTCCTATTATTAA 60

vrn-B3 ATGCTTTCGCTTGCCATCCCATCTCAGGAAACGTTCGCCAGACAGCATTCCTATTATTAA 60

Vrn-B3L TTTGTTTCAATATGATTTTTGCAGCTCATACCTTTGGAATAAAAAGAATCCAAAGGAAAA 120

Vrn-B3b TTTGTTTCAATATGATTTTTGCAGCTCATACCTTTGGAATAAAAAGAATCCAAAGGAAAA 120

vrn-B3 TTTGTTTCAATATGATTTTTGCAGCTCATACCTTTGGAATAAAAAGAATCCAAAGGAAAA 120

Vrn-B3L GATATAGGGTAGTGAAAAGATGCGCTCAAAGGGAACCAGGGCAACGAAAGCGGAGGGTAT 180

Vrn-B3b GATATAGGGTAGTGAAAAGATGCGCTCAAAGGGAACCAGGGCAACGAAAGCGGAGGGTAT 180

vrn-B3 GATATAGGGTAGTGAAAAGATGCGCTCAAAGGGAACCAGGGCAACGAAAGCGGAGGGTAT 180

Vrn-B3L ATTAAAAAGAAATTCAAAAACAATCAGAGGGTTTATTTCGAGCCCAGGGACAGGTGGAGC 240

Vrn-B3b ATTAAAAAGAAATTCAAAAACAATCAGAGGGTTTATTTCGAGCCCAGGGACAGGTGGAGC 240

vrn-B3 ATTAAAAAGAAATTCAAAAACAATCAGAGGGTTTATTTCGAGCCCAGGGACAGGTGGAGC 240

Vrn-B3L ATTATCATCGTCCTTGAGCCGGGACCGGCACTGTATATATATTCTCGCGGGAAAATGATT 300

Vrn-B3b ATTATCATCGTCCTTGAGCCGGGACCGGCACTGTATATATATTCTCGCGGGAAAATGATT 300

vrn-B3 ATTATCATCGTCCTTGAGCCGGGACCGGCACTGTATATATATTCTCGCGGGAAAATGATT 300

Vrn-B3L GTGGGGGGCTTTTTTCCTAATACGGCCCGCGTCCCGTGTAAGCTAACGTTTCGGGAAAGT 360

Vrn-B3b GTGGGGGGCTTTTTTCCTAATACGGCCCGCGTCCCGTGTAAGCTAACGTTTCGGGAAAGT 360

vrn-B3 GTGGGGGGCTTTTTTCCTAATACGGCCCGCGTCCCGTGTAAGCTAACGTTTCGGGAAAGT 360

Vrn-B3L CCGTCTTCTTCATCCTTTCCGACCACGCACGCAACCAGCAGCAAGAATGAAACCTGTCCT 420

Vrn-B3b CCGTCTTCTTCATCCTTTCCGACCACGCACACAACCAGCAGCAAGAATGAAACCTGTCCT 420

vrn-B3 CCGTCTTCTTCATCCTTTCCGACCACGCACACAACCAGCAGCAAGAATGAAACCTGTCCT 420

Vrn-B3L CCAATGATCTCCCC-GTACGACCCATCCGAGGCTGTGTGATCTTGCTCTCCCTCCCCGTC 480

Vrn-B3b CCAATGATCTCCCCCGTACGACCCATCCGAGGCTGTGTGATCTTGCTCTCCCTCCCCGTC 480

vrn-B3 CCAATGATCTCCCCCGTACGACCCATCCGAGGCTGTGTGATCTTGCTCTCCCTCCCCGTC 480

Vrn-B3L GTCACCATGCACGTGAAACGGCCGGGCTTTCGACACCTTCCCAGCTACGGCCGGCGGCAG 540

Vrn-B3b GTCACCATGCACGTGAAACGGCCGGGCTTTCGACACCTTCCCAGCTACGGCCGGCGGCAG 540

vrn-B3 GTCACCATGCACGTGAAACGGCCGGGCTTTCGACACCTTCCCAGCTACGGCCGGCGGCAG 540

Vrn-B3L CTGATGAAGCTTACATCAAATCGAGCCAAGGAAGCCTGCACCCCAGTCACCGTCGGCCGC 600

Vrn-B3b CTGATGAAGCTTACATCAAATCGAGCCAAGGAAGCCTGCACCCCAGTCACCGTCGGCCGC 600

vrn-B3 CTGATGAAGCTTACATCAAATCGAGCCAAGGAAGCCTGCACCCCAGTCACCGTCGGCCGC 600

Vrn-B3L TAGCTAATTGGCAGACATTCCCTGTGCCCGCTTGCCGGCCGGCCGCGGCGTGACCGCCGG 660

Vrn-B3b TAGCTAATTGGCAGACATTCCCTGTGCCCGCTTGCCGGCCGGCCGCGGCGTGACCGCCGG 660

vrn-B3 TAGCTAATTGGCAGACATTCCCTGTGCCCGCTTGCCGGCCGGCCGCGGCGTGACCGCCGG 660

Vrn-B3L TCGGCCCAGAGCCCCGGACGCAACGCAAACCTACACCCCAGCAGGCAGTGGCGGAGCCAG 720

Vrn-B3b TCGGCCCAGAGCCCCGGACGCAACGCAAACCTACACCCCAGCAGGCAGTGGCGGAGCCAG 720

vrn-B3 TCGGCCCAGAGCCCCGGACGCAACGCAAACCTACACC....................... 697

Vrn-B3L GAATATTTTTTAGGTGTGGCAGAGTTTGTGAGGGAAAAAAACAATGTAGTAACATATAAC 780

Vrn-B3b GAATATTTTTTAGGTGTGGCAGAGTTTGTGAGGGAAAAAAACAATGTAGTAACATATAAC 780

vrn-B3 ............................................................ 697

Vrn-B3L TATGTGACGTTCCTGCTTGCTGCCTCTCTTGACGGTGATCGCTGCCATGCCCACCACGCT 840

Vrn-B3b TATGTGACGTTCCTGCTTGCTGCCTCTCTTGACGGTGATCGCTGCCATGCCCACCACGCT 840

vrn-B3 ............................................................ 697

Vrn-B3L GACTAAAGATCGCGTGGTTGCTCTGTAATCGCTGCCTCTGTTGCATGCCTTACAGGTTAG 900

Vrn-B3b GACTAAAGATCGCGTGGTTGCTCTGTAATCGCTGCCTCTGTTGCATGCCTTACAGGTTAG 900

vrn-B3 ............................................................ 697

Vrn-B3L CCGACTAGGGGTGGGTCGGTCGGTCGGCAGTAGGGACAAATATCACAACAAGACATACAT 960

Vrn-B3b CCGACTAGGGGTGGGTCGGTCGGTCGGCAGTAGGGACAAATATCACAACAAGACATACAT 960

vrn-B3 ............................................................ 697

Vrn-B3L TGTCATGAGTTTGGAGGAGTAGCCGTTGCTTCTAGTTGTATATTGTAATACCTCAGACGA 1020

Vrn-B3b TGTCATGAGTTTGGAGGAGTAGCCGTTGCTTCTAGTTGTATATTGTAATACCTCAGACGA 1020

vrn-B3 ............................................................ 697

Vrn-B3L TTTGAGTCCGATTAGATCTAGTTTCTTCACCGGAGAGACGGAAAGCGGTGAGGCTCTGGG 1080

Vrn-B3b TTTGAGTCCGATTAGATCTAGTTTCTTCACCGGAGAGACGGAAAGCGGTGAGGCTCTGGG 1080

vrn-B3 ............................................................ 697

Vrn-B3L TTTCGCGGAGTTGGCACTGCTCATCACACAGCTAGAGACAAGCTTGAGGGGCGGCTGCAA 1140

Vrn-B3b TTTCGCGGAGTTGGCACTGCTCATCACACAGCTAGAGACAAGCTTGAGGGGCGGCTGCAA 1140

vrn-B3 ............................................................ 697

Vrn-B3L GGCGATTCCTGATCAACCGTGCTTGAACGAAACAGCTCGTGTATCTCCAATCAATTAGTA 1200

Vrn-B3b GGCGATTCCTGATCAACCGTGCTTGAACGAAACAGCTCGTGTATCTCCAATCAATTAGTA 1200

vrn-B3 ............................................................ 697

Vrn-B3L CATTTTTTTAGCATGTCCAATCAACTGGAGTACATGTGAATTGATTTGCAATTGACTGCC 1260

Vrn-B3b CATTTTTTTAGCATGTCCAATCAACTGGAGTACATGTGAATTGATTTGCAATTGACTGCC 1260

vrn-B3 ............................................................ 697

Vrn-B3L AATGGTGTCCTCGTTTAGAACATGGGCTTGTGGGTCATGGGCTTATGTGATGCAAGTCAC 1320

Vrn-B3b AATGGTGTCCTCGTTTAGAACATGGGCTTGTGGGTCATGGGCTTATGTGATGCAAGTCAC 1320

vrn-B3 ............................................................ 697

Vrn-B3L CTATTGCTTAATTCTCTCCTTCAGCGGCAGTTAGGCCTCTAATGAGGTAGAAACACGTAG 1380

Vrn-B3b CTATTGCTTAATTCTCTCCTTCAGCGGCAGTTAGGCCTCTAATGAGGTAGAAACACGTAG 1380

vrn-B3 ............................................................ 697

Vrn-B3L GTAACAACACAACTATCTGGCGAAATACATTTGAGGCCATGAGTTGCCTGCCCCCTGCGA 1440

Vrn-B3b GTAACAACACAACTATCTGGCGAAATACATTTGAGGCCATGAGTTGCCTGCCCCCTGCGA 1440

vrn-B3 ............................................................ 697

Vrn-B3L GTATTCATCCTCCAAATTGACGAAACTGACCAAAAATCCATTGACTCAGCTCAAAGCTTC 1500

Vrn-B3b GTATTCATCCTCCAAATTGACGAAACTGACCAAAAATCCATTGACTCAGCTCAAAGCTTC 1500

vrn-B3 ............................................................ 697

Vrn-B3L ATACATACAGTATATATATGTAATACTTTTGCCAAATTTTAGGTATGGCGGCTGCCAAAC 1560

Vrn-B3b ATACATACAGTATATATATGTAATACTTTTGCCAAATTTTAGGTATGGCGGCTGCCAAAC 1560

vrn-B3 ............................................................ 697

Vrn-B3L CTCGCCATATAGCTGGATCCGCCCCTGCCAGCAGGCACGGACAGAAACCACCATTAATTT 1620

Vrn-B3b CTCGCCATATAGCTGGATCCGCCCCTGCCAGCAGGCACGGACAGAAACCACCATTAATTT 1620

vrn-B3 ...........................CCAGCAGGCACGGACAGAAACCACCATTAATTT 730

Vrn-B3L GCGTGGTGATCATGATCAGGAGCTTATTACGGCAGACAGATGCATCCATCGGTCTCGCTT 1680

Vrn-B3b GCGTGGTGATCATGATCAGGAGCTTATTACGGCAGACAGATGCATCCATCGGTCTCGCTT 1680

vrn-B3 GCGTGGTGATCATGATCAGGAGCTTATTACGGCAGACAGATGCATCCATCGGTCTCGCTT 790

Vrn-B3L CTGCCTGTGGGGGTCAAAAGCGCTGCCGGTTACACCACATCCACAGAACCAATTGTACAG 1740

Vrn-B3b CTGCCTGTGGGGGTCAAAAGCGCTGCCGGTTACACCACATCCACAGAACCAATTGTACAG 1740

vrn-B3 CTGCCTGTGGGGGTCAAAAGCGCTGCCGGTTACACCACATCCACAGAACCAATTGTACAG 850

Vrn-B3L AGGGAGGCGACGAGATTCCGTGGCCACGCCAGCTCGGCAGCGCCAAGGAGTACTAGAGCG 1800

Vrn-B3b AGGGAGGCGACGAGATTCCGTGGCCACGCCAGCTCGGCAGCGCCAAGGAGTACTAGAGCG 1800

vrn-B3 AGGGAGGCGACGAGATTCCGTGGCCACGCCAGCTCGGCAGCGCCAAGGAGTACTAGAGCG 910

Vrn-B3L GCGAGCAGCGGCTGAACTGGTCTGGACATGGACATGTACCCTGCGTGAGCTTCTCGGCCC 1860

Vrn-B3b GCGAGCAGCGGCTGAACTGGTCTGGACATGGACATGTACCCTGCGTGAGCTTTTCGGCCC 1860

vrn-B3 GCGAGCAGCGGCTGAACTGGTCTGGACATGGACATGTACCCTGCGTGAGCTTTTCGGCCC 970

Vrn-B3L TATATAAAGTGGCCACCGGCCGTGGGGCAACACTCATCATCACCACTTCCTCAATTCACA 1920

Vrn-B3b TATATAAAGTGGCCACCGGCCGTGGGGCAACACTCATCATCACCACTTCCTCAATTCACA 1920

vrn-B3 TATATAAAGTGGCCACCGGCCGTGGGGCAACACTCATCATCACCACTTCCTCAATTCACA 1030

Vrn-B3L GCTTACTCCTGCTCCAGAGAACTTCTGCCTGCTGCCTCGTACCCTAGCTAGCAAGGCAAG 1980

Vrn-B3b GCTTACTCCTGCTCCAGAGAACTTCTGCTTGCTGCCTCGTACCCTAGCTAGCAAGGCAAG 1980

vrn-B3 GCTTACTCCTGCTCCAGAGAACTTCTGCTTGCTGCCTCGTACCCTAGCTAGCAAGGCAAG 1090

Vrn-B3L CTAGCCGGTCGATCTATACTAGGAAGGAAGGGCTA 2015

Vrn-B3b CTAGCCGGTCGATCTATACTAGGAAGGAAGGGCTA 2015

vrn-B3 CTAGCCGGTCGATCTATACTAGGAAGGAAGGGCTA 1125
