# Supplementary material for: Exploring and Mobilizing the Gene Bank Biodiversity for Wheat Improvement
Source: PLoS One. 2015 Jul 15;10(7):e0132112. doi: 10.1371/journal.pone.0132112 (PMC4503568; doi:10.1371/journal.pone.0132112)
Supplement: S2 Table — (DOCX) [file pone.0132112.s010.docx]

Table S2 Nei’s diversity index (DI) in landraces (FIGS Drought and Australia Hot), synthetic hexaploids and elite lines using 211 samples in each group

| LG | FD | AH | SH | E |  |
| --- | --- | --- | --- | --- | --- |
| 1A | 0.241 | 0.264 | 0.252 | 0.313 |  |
| 1B | 0.272 | 0.272 | 0.314 | 0.261 |  |
| 1D | 0.253 | 0.275 | 0.333 | 0.264 |  |
| 2A | 0.233 | 0.256 | 0.287 | 0.255 |  |
| 2B | 0.243 | 0.264 | 0.265 | 0.296 |  |
| 2D | 0.205 | 0.182 | 0.317 | 0.224 |  |
| 3A | 0.222 | 0.264 | 0.265 | 0.276 |  |
| 3B | 0.232 | 0.285 | 0.264 | 0.286 |  |
| 3D | 0.173 | 0.195 | 0.384 | 0.196 |  |
| 4A | 0.284 | 0.273 | 0.274 | 0.284 |  |
| 4B | 0.212 | 0.223 | 0.304 | 0.255 |  |
| 4D | 0.194 | 0.172 | 0.386 | 0.243 |  |
| 5A | 0.233 | 0.275 | 0.244 | 0.287 |  |
| 5B | 0.263 | 0.263 | 0.275 | 0.306 |  |
| 5D | 0.193 | 0.225 | 0.317 | 0.204 |  |
| 6A | 0.261 | 0.294 | 0.283 | 0.295 |  |
| 6B | 0.283 | 0.284 | 0.253 | 0.305 |  |
| 6D | 0.285 | 0.284 | 0.385 | 0.314 |  |
| 7A | 0.222 | 0.245 | 0.287 | 0.266 |  |
| 7B | 0.253 | 0.314 | 0.295 | 0.316 |  |
| 7D | 0.224 | 0.212 | 0.394 | 0.172 |  |
| Mean | 0.237 | 0.253 | 0.303 | 0.267 |  |

LG: Linkage group; FD: FIGS Drought; AH: Australia Hot; SH: Synthetic hexaploids; E: Elite
