# Supplementary material for: Exploring and Mobilizing the Gene Bank Biodiversity for Wheat Improvement
Source: PLoS One. 2015 Jul 15;10(7):e0132112. doi: 10.1371/journal.pone.0132112 (PMC4503568; doi:10.1371/journal.pone.0132112)
Supplement: S3 Table — (DOCX) [file pone.0132112.s011.docx]

Table S3 Thirty nine allele specific gene-based SNP and STS markers used in the present study

| Gene allele | Trait | STS/SNP* |
| --- | --- | --- |
| *RhtB1b* | Height | SNP |
| *RhtD1b* | Height | SNP |
| *Fhb1* | Fusarium head blight | SNP |
| *LR34* | Leaf rust | SNP |
| 1RS:1BL | Leaf rust, stripe rust, stem rust, powdery mildew | SNP |
| *GluA1* | HMW subunits of glutenin | SNP |
| *GluD1* (5+10) | HMW subunits of glutenin | SNP |
| *Sr36* | Stem rust | SNP |
| *Sbm1* | Soil born mosaic virus | SNP |
| *GPC* | Grain protein content | SNP |
| *PinbD1b* | Grain hardness | SNP |
| *PinaD1b* | Grain hardness | SNP |
| *PpdD1a* | Flowering | SNP |
| *PpdA1a* (GS 100 type deletion) | Flowering | SNP |
| *Psy 1 D1* | Yellow pigment | SNP and STS |
| *VrnA1a* | Flowering | STS and STS |
| *VrnA1b* | Flowering | SNP |
| *VrnA1c* | Flowering | STS |
| *VrnB1a* | Flowering | STS |
| *VrnB1b* | Flowering | SNP |
| *VrnD1* | Flowering | STS |
| *GluA3f* | LMV subunits of glutenin | STS |
| *GluA3b* | LMV subunits of glutenin | STS |
| *GluA3g* | LMV subunits of glutenin | STS |
| *GluA3ac* | LMV subunits of glutenin | STS |
| *GluB3b* | LMV subunits of glutenin | STS |
| *GluB3i* | LMV subunits of glutenin | STS |
| *Gene allele* | Trait | STS/SNP* |
| *GluB3d* | LMV subunits of glutenin | STS |
| *GluB3bef* | LMV subunits of glutenin | STS |
| *GluB3fg* | LMV subunits of glutenin | STS |
| *Pm3a* | Powdery mildew | STS |
| *Pm3b* | Powdery mildew | STS |
| *Pm3c* | Powdery mildew | STS |
| *Pm3d* | Powdery mildew | STS |
| *Pm3e* | Powdery mildew | STS |
| *Pm3f* | Powdery mildew | STS |
| *Pm3g* | Powdery mildew | STS |
| *VpB1* | Preharvest sprouting tolerance | STS |
| *PPO33* | Wheat flour whiteness | STS |

*Markers and primers reported on MASwheat and CerealsdB databases
