# Supplementary material for: Exploring and Mobilizing the Gene Bank Biodiversity for Wheat Improvement
Source: PLoS One. 2015 Jul 15;10(7):e0132112. doi: 10.1371/journal.pone.0132112 (PMC4503568; doi:10.1371/journal.pone.0132112)
Supplement: S4 Table — (DOCX) [file pone.0132112.s012.docx]

**Table S4:** Allele frequencies of gene alleles in the landrace germplasm from different countries

| Gene |  |  | Country |  |  |
| --- | --- | --- | --- | --- | --- |
|  | Afghanistan | India | Iran | Iraq | Pakistan |
| *LR34* | 0.043 | 0.000 | 0.005 | 0.000 | 0.000 |
| *GluA1* | 0.014 | 0.024 | 0.016 | 0.136 | 0.037 |
| *Glu D1* 5+10 | 0.014 | 0.049 | 0.031 | 0.227 | 0.057 |
| *Sbm1* | 0.096 | 0.928 | 0.947 | 0.727 | 0.945 |
| *Pinb_D1b* | 0.021 | 0.047 | 0.037 | 0.090 | 0.040 |
| *Pina_D1b* | 0.007 | 0.381 | 0.010 | 0.286 | 0.067 |
| *PpdD1a* | 0.006 | 0.000 | 0.005 | 0.000 | 0.009 |
| *Psy1 D1^+^* | 0.007 | 0.000 | 0.016 | 0.000 | 0.018 |
| *Psy1 D1^++^* | 0.020 | 0.000 | 0.047 | 0.000 | 0.000 |
| *VrnA1a* | 0.027 | 0.070 | 0.069 | 0.042 | 0.065 |
| *VrnA1b* | 0.000 | 0.023 | 0.026 | 0.136 | 0.009 |
| *VrnD1* | 0.957 | 0.958 | 0.978 | 0.400 | 0.984 |
| *VRN A1c* | 0.253 | 0.204 | 0.097 | 0.000 | 0.009 |
| *VrnB1a* | 0.072 | 0.045 | 0.157 | 0.600 | 0.189 |
| *GluA3-f* | 0.007 | 0.000 | 0.011 | 0.047 | 0.037 |
| *GluA3-b* | 0.662 | 0.931 | 0.952 | 0.920 | 0.910 |
| *GluA3-g* | 0.728 | 0.272 | 0.069 | 0.080 | 0.175 |
| *GluA3-ac* | 0.666 | 0.666 | 0.631 | 0.739 | 0.816 |
| *GluB3-b* | 0.264 | 0.227 | 0.779 | 0.400 | 0.752 |
| *GluB3 – i* | 0.351 | 0.113 | 0.479 | 0.080 | 0.288 |
| *GluB3-d* | 0.103 | 0.488 | 0.084 | 0.208 | 0.296 |
| *GluB3-bef* | 0.059 | 0.045 | 0.684 | 0.000 | 0.409 |
| *GluB3-fg* | 0.337 | 0.159 | 0.239 | 0.200 | 0.089 |
| *PM3-b* | 0.026 | 0.000 | 0.036 | 0.000 | 0.000 |
| *PM3-c* | 0.013 | 0.090 | 0.052 | 0.080 | 0.017 |
| *PM3-f* | 0.000 | 0.022 | 0.026 | 0.000 | 0.053 |
| *PM3-g* | 0.000 | 0.000 | 0.721 | 0.000 | 0.715 |
| *VpB1* | 0.148 | 0.627 | 0.054 | 0.652 | 0.480 |
| *PPO33* | 0.425 | 0.090 | 0.131 | 0.083 | 0.234 |

*^Psy 1 D1+ =^* ^Allele for high yellow pigment;^

*^Psy 1 D1++ =^* ^Allele for low yellow pigment^
